# Large-Scale Nonequilibrium Molecular Studies of Thermal Hydrate Dissociation

**DOI:** 10.1021/acs.jpcb.3c03391

**Published:** 2023-07-18

**Authors:** Meisam Adibifard, Olufemi Olorode

**Affiliations:** † Department of Petroleum Engineering, Louisiana State University, Baton Rouge, Louisiana 70803, United States

## Abstract

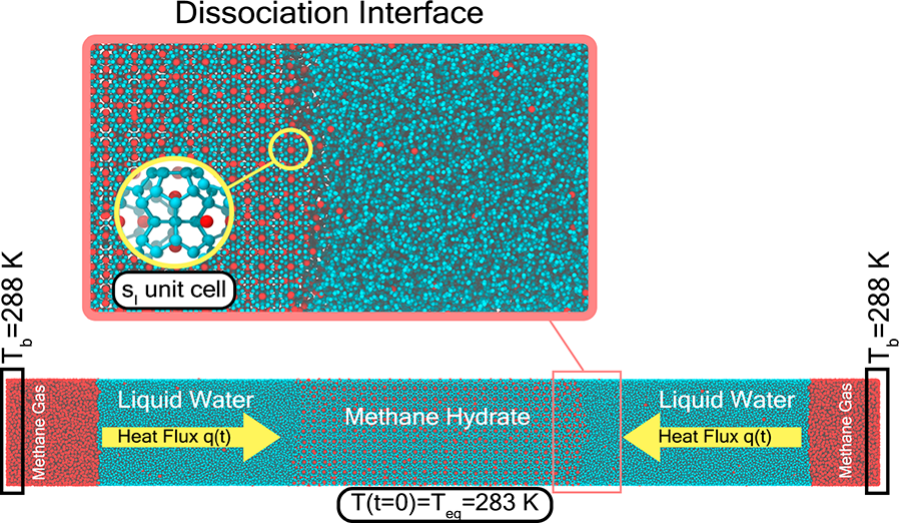

The energy content of methane hydrate reservoirs (MHRs) is at least twice that of conventional fossil fuels. So, there is considerable interest in their commercial development by heating, among other dissociation mechanisms. However, a few researchers have highlighted the potentially uncontrollable release of methane from MHRs, which could occur because of global warming. Therefore, it is crucial to understand the kinetics of thermal hydrate dissociation to safely develop these resources and prevent the release of this greenhouse gas into the environment. Although there have been several molecular studies of thermal dissociation, most of these use small simulation domains that cannot capture the transient nature of the process. To address this limitation, we performed coarse-grained molecular dynamics (CGMD) simulations on a significantly larger domain with a hundred times more hydrate unit cells than those used in previous studies. We monitored the kinetics of dissociation using an image-processing algorithm and observed the dynamics of the process while maintaining a thermal gradient at the dissociation front. For the first time, we report the formation of an unstable secondary dissociation path that triggers gas bubbles within the solid hydrate. The kinetics of thermal dissociation appears to occur in three stages. In the first stage, the energy of the system increases until it exceeds the activation energy, and dissociation is initiated. Consistent dissociation occurs in the second stage, whereas the third stage involves the dissociation of the remaining hydrates across a nonplanar and heterogeneous interface.

## Introduction

Gas hydrates are nonstoichiometric icelike compounds that form under high-pressure and low-temperature conditions, when guest (mostly gas) molecules are confined in the cavities of the crystal lattices formed by the host (water) molecules.^1,2^ They have been studied in the context of flow assurance, CO_2_ capture and storage (CCS), gas separation, desalination, refrigeration, and energy recovery.^3^ There is a consensus that the stored energy in natural gas hydrate (NGH) reservoirs is at least twice that of conventional hydrocarbon reservoirs.^4^ Hydrates have also been considered as potentially viable options for storing and transporting gases such as methane, carbon dioxide, and hydrogen^5−7^ because each cubic meter of the hydrates can hold 120–180 m^3^ of these gas molecules.^4^ The temperature, pressure, and nature of the guest molecules determine the structure of the gas hydrate. The three most common hydrate structures are structure I (s_I_), structure II (s_II_), and structure H (s_H_), each consisting of different types of cages.^8,9^

Thermal dissociation involves heating gas hydrates in order to release and produce the gases trapped in them by breaking the hydrogen bonds between the water molecules. Considering that the dissociation and production of gases from hydrates by depressurization alone have not been demonstrated to be commercially viable,^10^ several authors^10−12^ have evaluated thermal dissociation either in isolation or in combination with depressurization. It is essential to accurately estimate the dissociation rate to facilitate the commercial development of NGH reservoirs by thermal dissociation. Although most reservoir-scale numerical dissociation studies use the model of Kim et al.^13^ to predict the mass rate of dissociation, the experiments used to develop this empirical model were performed at a constant temperature. Thus, the applicability of the model in estimating the mass rate of thermal dissociation at a fixed pressure is questionable.

Despite the considerable number of molecular studies on gas hydrate dissociation,^11,14−17^ the transient nature of hydrate dissociation at the molecular scale is still poorly understood^18^ because most of the previous molecular studies involve isothermal simulations. These isothermal simulations use a global thermal bath to modify the momentum of atoms.^8,15−17^ Consequently, they result in artificially increased mass transfer rates^19^ and cannot capture the expected thermal gradients at the hydrate/liquid interface during dissociation.^20^ Additionally, a few researchers^15,21−23^ have studied transient thermal dissociation using adiabatic ensembles, but the changes in the average pressure of the simulated systems indicate that these results incorporate the effect of the pressure variation in addition to the thermal dissociation being studied.

Furthermore, most of the previous studies used all-atom models for the host and guest molecules, which benefitted from using published model parameters that are applicable over a wide range of pressure and temperature conditions, but were limited to small simulation domains with a few hundred molecules. These small-length scales could yield statistically insignificant results because only a few hydrate cages are dissociated during the simulation of these small systems; there are also more fluctuations in the thermodynamic properties of the system. To address these limitations, we will study the kinetic process of transient thermal dissociation at constant pressure and at much larger scales than has been done in previous studies. Thus, we leverage the coarse-grained monatomic water (mW) model and a reparametrized united atom (UA) model for methane.^24^ The mW model has the advantage of being up to three orders of magnitude faster than all-atom models with Ewald sums,^25^ though it requires tuning for different atoms/molecules, pressure conditions, and temperature conditions.

## Materials and Methods

### Force Field

We used the Large-scale Atomic-Molecular Massively Parallel Simulator (LAMMPS) to perform the simulations. We used the following reparametrized form of the Stillinger–Weber (SW) potential^25^ for force field calculations:
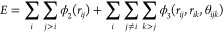
1
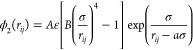

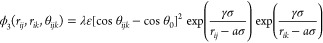
Here, ϕ_2_(*r*_*ij*_) and ϕ_3_(*r*_*ij*_, *r*_*ik*_, *θ*_*ijk*_) are two-body and three-body interaction terms, respectively. The idea of this model is to mimic the hydrogen-bonding structure of water by adding ϕ_3_, which is a penalty term that encourages the tetrahedral configuration of water. The two critical parameters of the model are the size scale (σ) and the energy scale (ε). The symbol *r*_*ij*_ represents the distance between the *i*th and *j*th particles, whereas *θ*_*ijk*_ is the angle between the *i*–*j* and *i*–*k* position vectors. The constants *A*, *B*, γ, λ, *a*, and θ_0_ are reported by Jacobson and Molinero^25^ as follows:
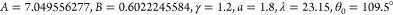


The interaction term (λ) is set to zero for all pairs of molecules, such as water–water, methane–methane, and water–methane. Thus, the three-body interaction term is calculated only for the water–water–water interactions. All intermolecular potentials go to zero at the cutoff distance. Jacobson and Molinero^25^ matched the simulation results with the experimental data and found the optimal values of σ and ε for water, methane, and water–methane. They also pointed out the model’s applicability in estimating the relevant physical properties of the hydrate, water, and methane system.

### Initial Configuration

**Figure 1:**
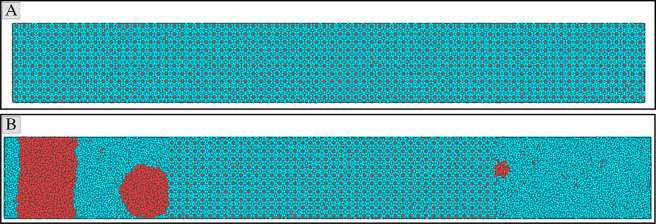
Initial hydrate crystal and the equilibrated hydrate/water/methane mixture. Image (A) shows the hydrate crystals at 100 atm and 250 K, whereas image (B) shows the hydrate/water/methane mixture under the hydrate equilibrium conditions (at 100 atm and 283 K). The cyan and red spheres represent the water and methane molecules, respectively. The larger size of the simulation box in image (B) compared to that in image (A) can be attributed to the formation and growth of the gas bubbles after melting the hydrate.

We populated the simulation box with 80 × 10 × 10 s_I_ unit cells of a methane hydrate in the *x*-, *y*-, and *z*-directions, resulting in a total of 432 000 atoms and an initial dimension of 96 × 12 × 12 nm. To equilibrate the hydrate crystals at conditions where the hydrate is stable, we performed NVT equilibration runs at a temperature of 250 K for 500 ps, followed by NPT simulations at the same temperature and a pressure of 100 atm for 1.0 ns. The methane hydrate under these conditions is used to create the equilibrium configuration for the thermal dissociation simulation. This system, shown in Figure 1A, is two orders of magnitude larger than most previous studies.^8,11,26,27^

To obtain the equilibrium configuration for the dissociation simulations, we partially melted the stabilized hydrate crystals and calculated the equilibrium temperature (also referred to as the dissociation or melting temperature) using the direct coexistence method.^28^ The hydrate box was divided into four quarters in the *x*-direction. The first and last quarters were then subjected to an NVT simulation for 10 ns at the dissociation temperature of 400 K, which is much higher than the reported equilibrium temperature of 286.2 K at 100 atm.^25^ During the NVT simulation, the hydrate crystals in the middle of the box were fixed under the initial condition.

**Figure 2:**
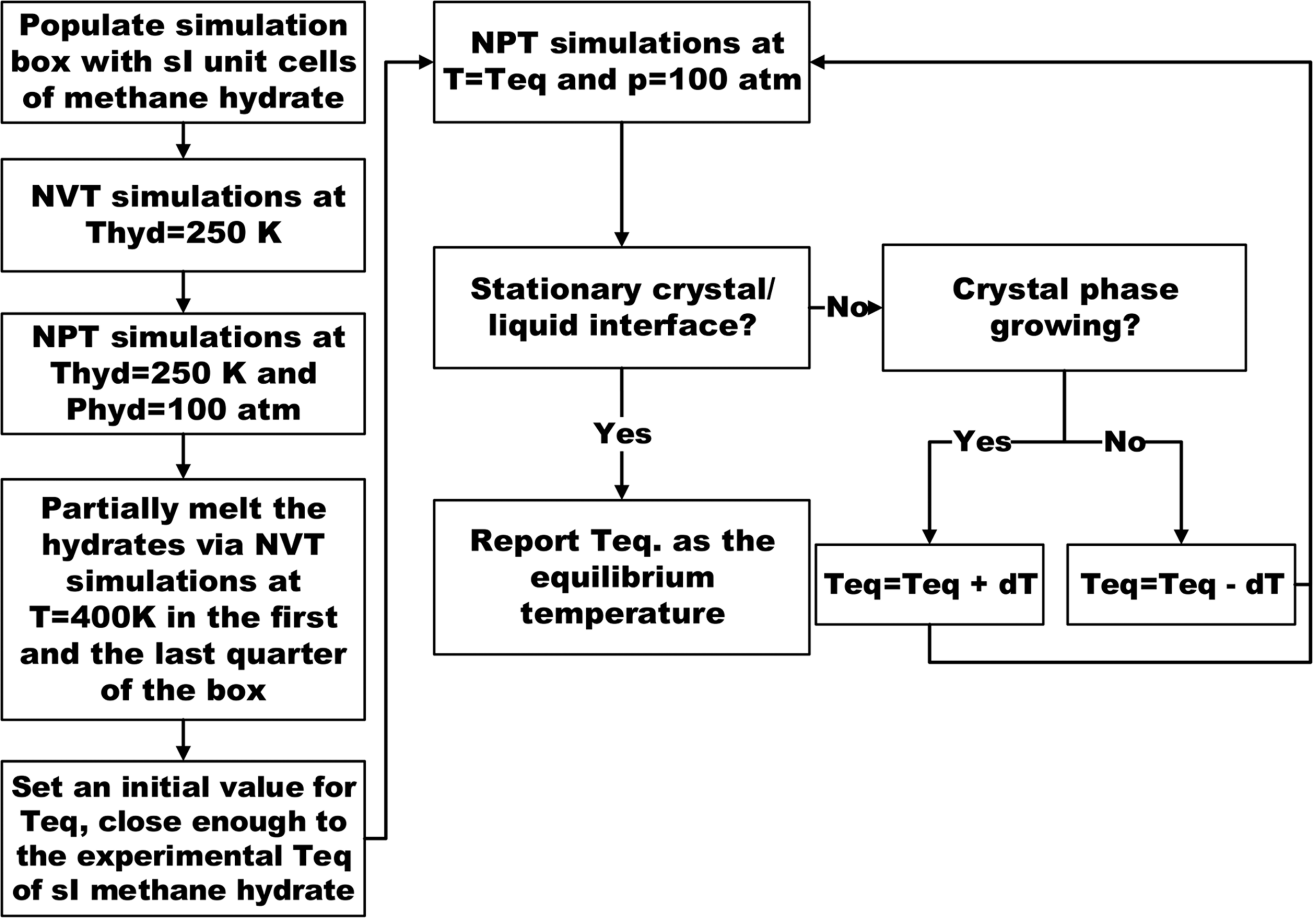
A flowchart that illustrates the simulation procedure used to calculate the equilibrium temperature of s_I_ methane hydrates and to obtain the initial configuration for the thermal dissociation simulations.

Figure 2 presents a flowchart that describes a series of system-wide NPT simulations that we performed at 100 atm and at varying temperatures in order to find the equilibrium temperature and state of the simulation box. We varied the temperature between 275 ± 290 K using an increment of 1 K and estimated the equilibrium temperature of methane hydrate to be 281.5 ± 1.5 K. When compared to the predicted value of 285 ± 4 K by Jacobson et al.,^25^ it can be seen that our estimated range of equilibrium temperature overlaps with that of Jacobson et al.^25^ The difference in the mean of the equilibrium temperatures might be attributed to the difference in size of the studied systems. We used an upper equilibrium temperature of 283 K for the thermal dissociation simulations presented in this work. The attained equilibrium configuration is shown in Figure 1B.

### Transient Thermal Dissociation

To simulate boundary-driven thermal dissociation, we performed isenthalpic–isobaric (NPH) simulations with a stochastic Langevin thermostat (on the left and right boundaries of the simulation box), starting with the equilibrated three-phase system. The Langevin thermostat was maintained at a boundary temperature greater than the equilibrium temperature (*T*_b_ > *T*_eq_) by using a damping factor of 0.1 ps. The higher boundary temperature generates a symmetric thermal gradient that propagates from the left and right boundaries toward the interior of the simulation box. We repeated these NPH simulations at different magnitudes of *T*_b_.

To estimate the mass of the solid hydrates left in the simulation box, we implemented a template-matching algorithm^29^ to track the number of hydrate cages in the simulation box. This, coupled with the known mass of each hydrate cage, can be used to estimate the mass rate of dissociation over the simulated period. Considering that there are several hydrate cages in each direction and that the dissociation rate is not constant across all *xy*-plane slices of the simulation box, we generated *N*_*z*_ slices of images in the *xy*-plane and at several output time steps. From these 2D images of the sliced trajectories in the *xy*-plane, the mass of the remaining hydrate is estimated as
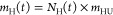
2where *m*_H_(*t*) is the mass of hydrate, *N*_H_(*t*) is the number of hydrate unit cells, and *m*_HU_ is the mass per hydrate unit cell, which is estimated as
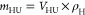
3where *V*_HU_ is the volume of a hydrate unit cell (*V*_HU_ = 1.728 nm^3^) and *ρ*_H_ is the density of the hydrate (0.9 g/cm^3^). As expected, *N*_H_(*t*) varies with simulation time and is calculated as follows:
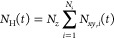
4where *N*_*xy,i*_(*t*) is the number of hydrate crystals in the *xy*-plane of the *i*th slice at time *t*; it is estimated using the template-matching algorithm.^29^ Template matching is a method for determining the parts of an image that match a smaller repeating pattern or template image. Our implementation of the algorithm takes in the images of the top and bottom halves of a unit cell and counts the number of occurrences of each of these in any given cross-sectional area of the simulation domain at a given time step. The total number of unit cells in each image of a slice of the simulation domain, *N*_*xy,i*_(*t*), is estimated as follows:
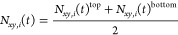
5Here, *N*_*xy,i*_(*t*)^top^ and *N*_*xy,i*_(*t*)^bottom^ are the numbers of top- and bottom-half instances of an s_I_ unit cell identified by the template-matching algorithm, respectively. The template-matching code is available in a GitHub repository (https://github.com/UnconvRS/ThermalDissociation). It is worth mentioning that a standard implementation using a complete unit cell instead of two half cells is less accurate because we typically have fractional unit cells at the top and bottom boundaries of the domain due to the bulk movement of the solid hydrate across these periodic boundaries. The instantaneous rate of dissociation per unit area can be calculated as the time derivative of the mass–time plot:
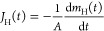
6

Here, *J*_H_(*t*) is the dissociation rate per unit area and *A* is the area of the dissociation front. The average *J*_H_ value can be estimated by fitting a straight line to the mass-time plot over the given interval.

## Results and Discussion

### Evolution of Thermal Dissociation at 288 K

**Figure 3:**
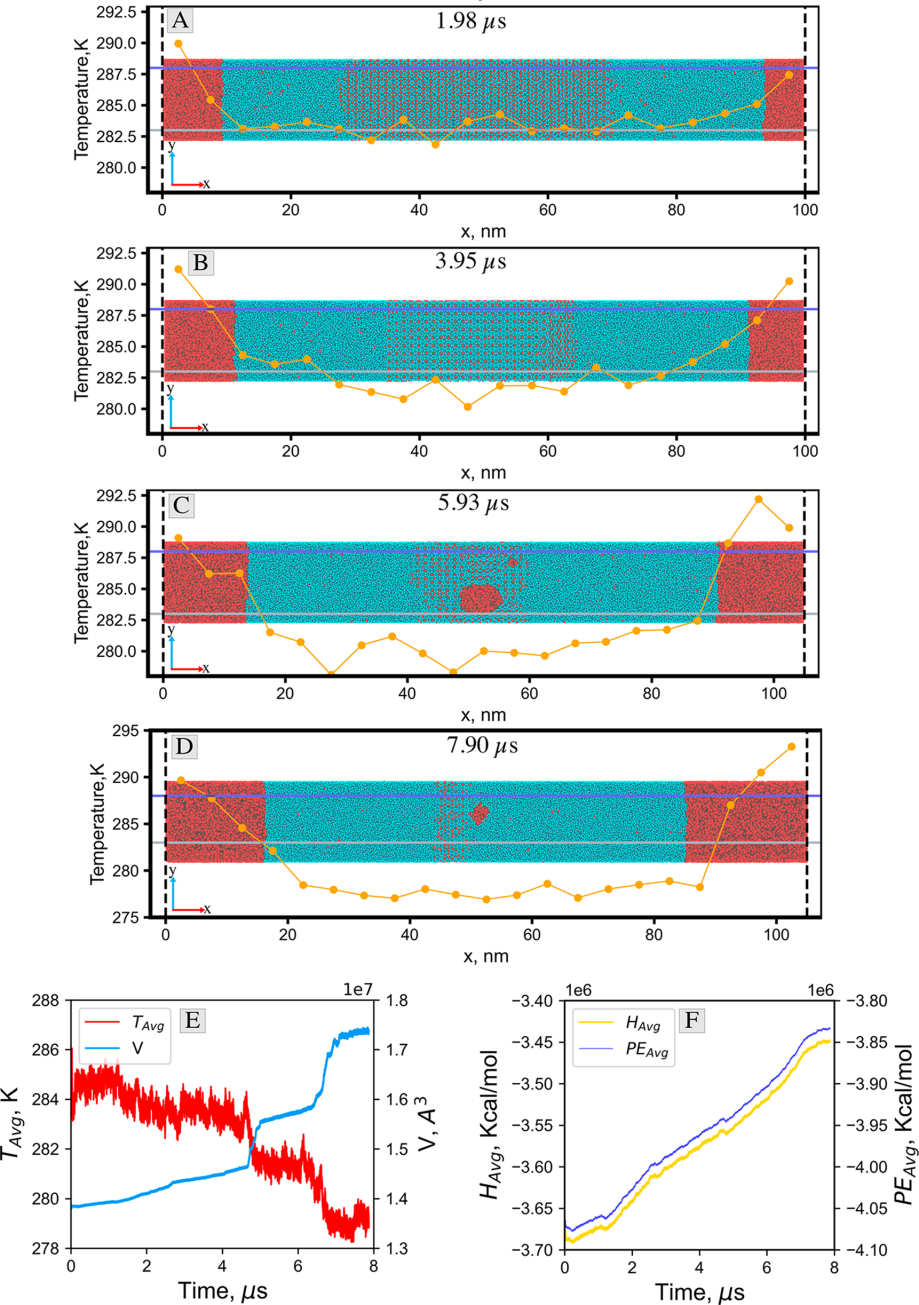
(A)–(D) show snapshots of the molecular trajectories overlaid with the temperature profile, and (E) and (F) show the evolution of the average temperature, volume, enthalpy, and potential energy of the system during the nonequilibrium dissociation simulations at *T*_b_ = 288 K. The snapshots represent the state of the system where (A) 25%, (B) 50%, (C) 75%, and (D) 100% of the simulation duration is completed. Water and methane are represented by cyan and red spheres, respectively. The orange line with solid circles represents the temperature profile, and the blue and gray horizontal lines represent the boundary temperature (*T*_b_ = 288 K) and the equilibrium temperature (*T*_eq_ = 283 K), respectively. The dotted vertical lines show the boundaries of the simulation box. Movie S1 provides a closer look into the crystal/liquid interface at the beginning of the dissociation, while Movie S2 illustrates the dynamics of the transient dissociation of the methane hydrate at a specified time interval.

Figure 3 presents the snapshots of the molecular trajectories overlaid with the temperature profile for nonequilibrium dissociation simulations at a boundary temperature of 288 K. To understand the evolution of the temperature in the system, we partitioned the simulation box into multiple slabs of 5 nm in the *x*-direction and computed the average temperature of each of these slabs. These average temperature values were then plotted against the position of the centroids of the slabs to obtain the 1D temperature profiles overlaid on the molecular snapshots in Figure 3. From the images in this figure, we observe that the temperature rises gradually from the left and right boundaries of the box toward the crystal/liquid interface, as heat is added to the system from these boundaries. The images presented in panels A and B of Figure 3 correspond to the periods in which the solid hydrate in the middle has dissociated to an extent, but there is no gas or liquid within the hydrate region. However, in the remaining images (where *t* > 4 μs), we observe some gas bubbles forming within the hydrate region.

The temperature profiles in panels A and B of Figure 3 show that the average temperature in the hydrate region and near the solid/liquid interface remains close to the equilibrium temperature of 283 K as the hydrate melts at the solid/liquid interface. This is consistent with the thermodynamics of melting and could indicate that the thermal energy that makes it to the interface is consumed by breaking the hydrogen bonds between the water molecules. Beyond the interface, the temperature rises gradually toward the boundary temperature of 288 K.

However, panels C and D of Figure 3 show that the temperature decreases further in the middle of the domain as the solid/liquid interface progresses toward the center of the box and that the hydrate is almost completely dissociated. This can be attributed to the rapid volume expansion of the simulation box (at a fixed pressure) because of the large volume of gas in the system toward the end of the simulation.

Additionally, the plots of the average temperature and the simulation box volume in Figure 3E show that the steep temperature drops are aligned with the corresponding sharp increases in the simulation box volume. For example, we observe abrupt changes in volume and temperature between 4.6 and 4.8 μs and between 6.1 and 7 μs. The rapid simulation box volume expansion and subsequent temperature drop toward the end of the simulation could be curtailed by increasing the value of the pressure-damping factor/parameter used in LAMMPS, as it essentially controls the time interval over which the volume is allowed to change in order to relax the pressure. However, large pressure-damping factor values could lead to deviations from the target pressure of the system, which is inconsistent with our goal of simulating transient thermal dissociation at a constant pressure.

The enthalpy and potential energy (PE) of the system increase continuously and with a similar trend during the thermal dissociation, as seen in Figure 3F. Figure S1 presents the corresponding results for the *T*_b_ = 293 K case.

### Nonplanarity of the Solid/Liquid Interface and Formation of a Secondary Dissociation Path

**Figure 4:**
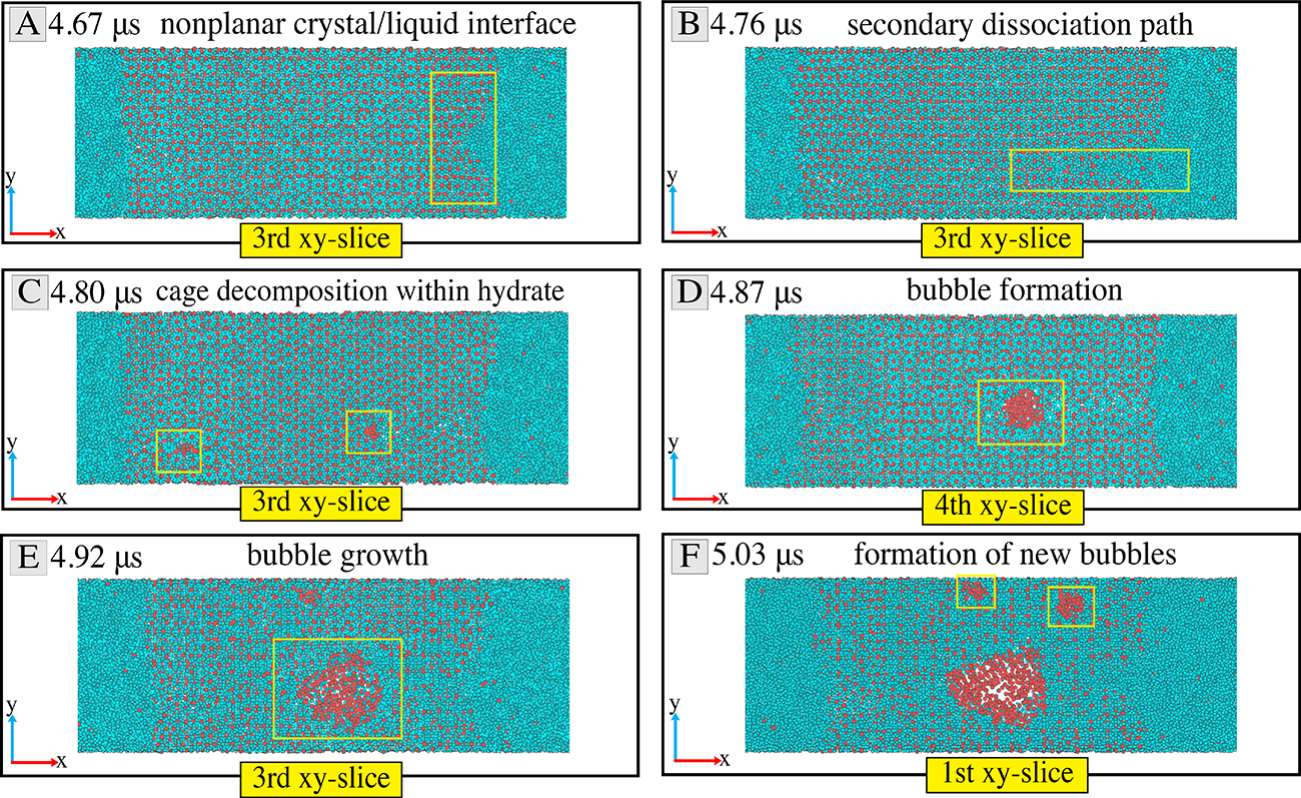
Molecular trajectories in different *xy*-slices representing the formation of a secondary dissociation path and bubble generation within the solid hydrate. These snapshots illustrate (A) nonplanarity of the dissociation front, (B) formation of the secondary dissociation path, (C) the onset of cage decomposition within the solid hydrate, (D) bubble formation in the solid hydrate, (E) bubble growth, and (F) an increase in the number of gas bubbles in the hydrate at *T*_b_ = 288 K. We selected the fourth and first *xy*-slices in (D) and (F), respectively, because the entire solid hydrate is translated as a rigid body across the periodic boundary. So, this translation caused the bubbles observed in the third slice in (C) to move into the fourth slice in (D), back to the third slice in (E), and into the first slice in (F). These translations of the location of the gas bubbles can be observed in the video of all five slices in Movie S3. Movie S4 provides a closer look into the third plane for better visualization of the channelized path of dissociation.

Considering that Figure 3 shows only the front view (or first *xy*-slice) of the simulation box, we visualized several *xy*-slices of the simulation box to reveal the internal structure of the hydrates as the simulation evolves. Each *xy*-slice consists of two hydrate cages in the *z*-direction, normal to the *xy*-plane. Figure 4 presents a couple of these slices at different time steps for the thermal dissociation at 288 K. Figure 4A shows the formation of a nonplanar dissociation front due to the non-uniformity of the dissociation rate at the interface. This observation indicates that the solid/liquid interface is not perfectly vertical during the thermal dissociation of these hydrates. More interestingly, the non-uniform dissociation rate leads to a channelized dissociation path in Figure 4B. As this secondary dissociation path penetrates the solid hydrate, it causes the decomposition of the hydrate cages and a consequent release of methane molecules trapped in them. Although some of the released methane gas dissolves into the liquid phase, some remains in the hydrate, as seen in Figure 4C. We observe from this image that the secondary dissociation path is unstable and fades away due to the reformation of hydrates.

Figure 4D shows the fourth *xy*-slice instead of the third one because the translation of the entire solid hydrate across the periodic boundary translates the gas bubble into the fourth slice. The observation of a larger bubble in Figure 4D, despite the reformation of hydrates that eliminates the secondary dissociation path, indicates that the methane gas molecules in the solid hydrate exist as stable bubbles. The time interval between the formation of the secondary dissociation path and the formation of the bubbles within the solid hydrate is ∼0.11 μs. Figure 4E shows a significant growth in the gas bubble size within the solid hydrate and a translation of the location of the bubble back into the third *xy*-slice. As the simulation evolves, more bubbles form within the solid hydrate, as shown in Figure 4F. Figure S2 presents the corresponding results for the *T*_b_ = 293 K case.

To confirm that the observation of a secondary dissociation path and having gas bubbles in the hydrate is not unique to the cases simulated, we repeated these dissociation simulations with different initial velocities and still observed the secondary path and gas bubbles. It appears that our studies of much larger systems (compared to previous studies) enabled this new observation.

### Kinetics of Thermal Dissociation

**Figure 5:**
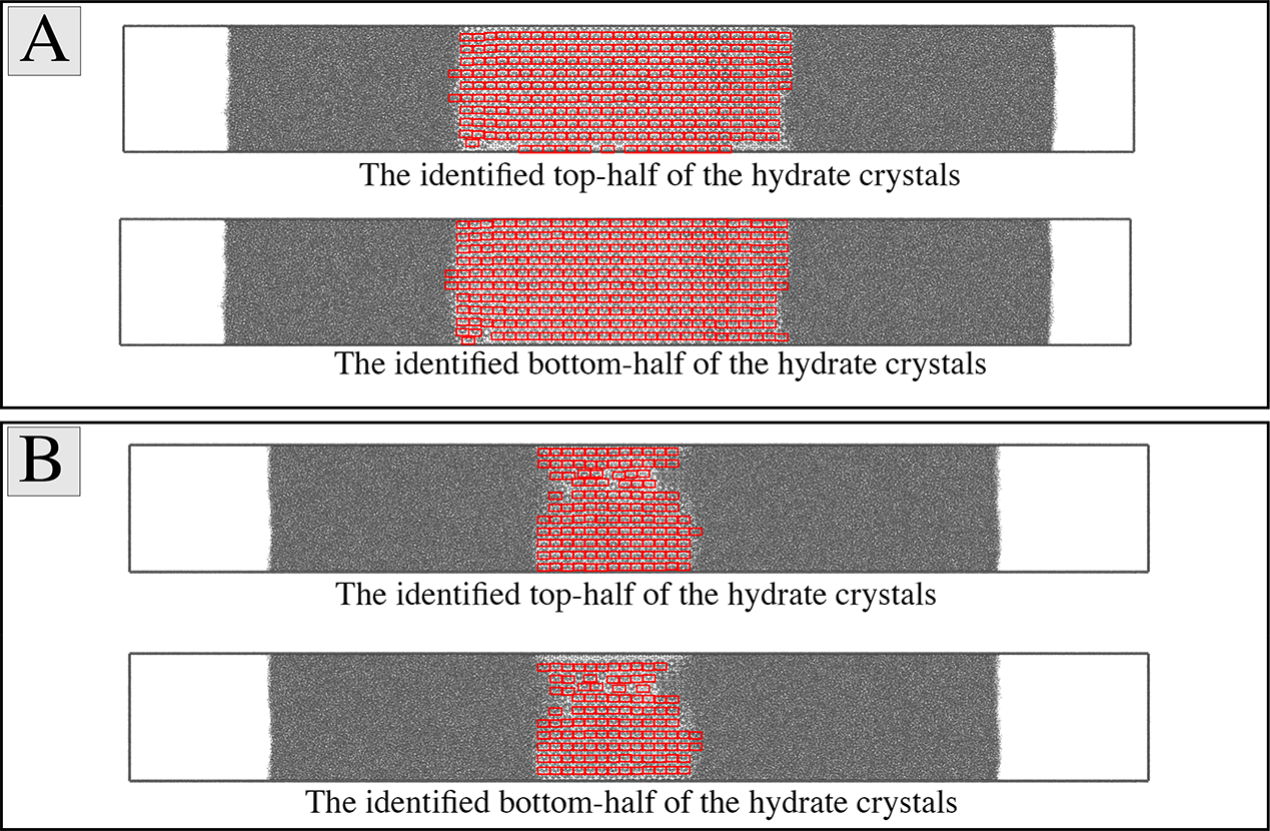
Images show the identified hydrate crystals using the template-matching algorithm with the NPH simulation results for the *T*_b_ = 288 K case at the (A) middle and (B) late stages of the simulation. The gray dots represent water molecules, and the identified crystals are bound with red rectangles. The methane molecules are omitted to facilitate the identification of the hydrate motifs.

To quantify the kinetics of methane hydrate dissociation, we counted the number of hydrate cages in the simulation domain at different time steps by providing the corresponding images of multiple *xy*-slices of the simulation box as inputs for the implemented template-matching code. We estimated the hydrate mass that remained in the simulation box by counting the remaining hydrate unit cells, as outlined in eqs 2–6. The identified hydrate cells are visualized in Figure 5 at two different simulation times. In order to assess the accuracy of the template-matching algorithm used to estimate the number of unit hydrate cells, we visually counted the number of unit cells in all five slices of the simulation domain (as shown in Figure 4) at five different simulation points over the simulated period. The errors in the template-matching algorithm at these points are summarized in Figure S3, which shows that the algorithm is accurate within 5% for four of the five points and less than 10% in the time step with the most error.

**Figure 6:**
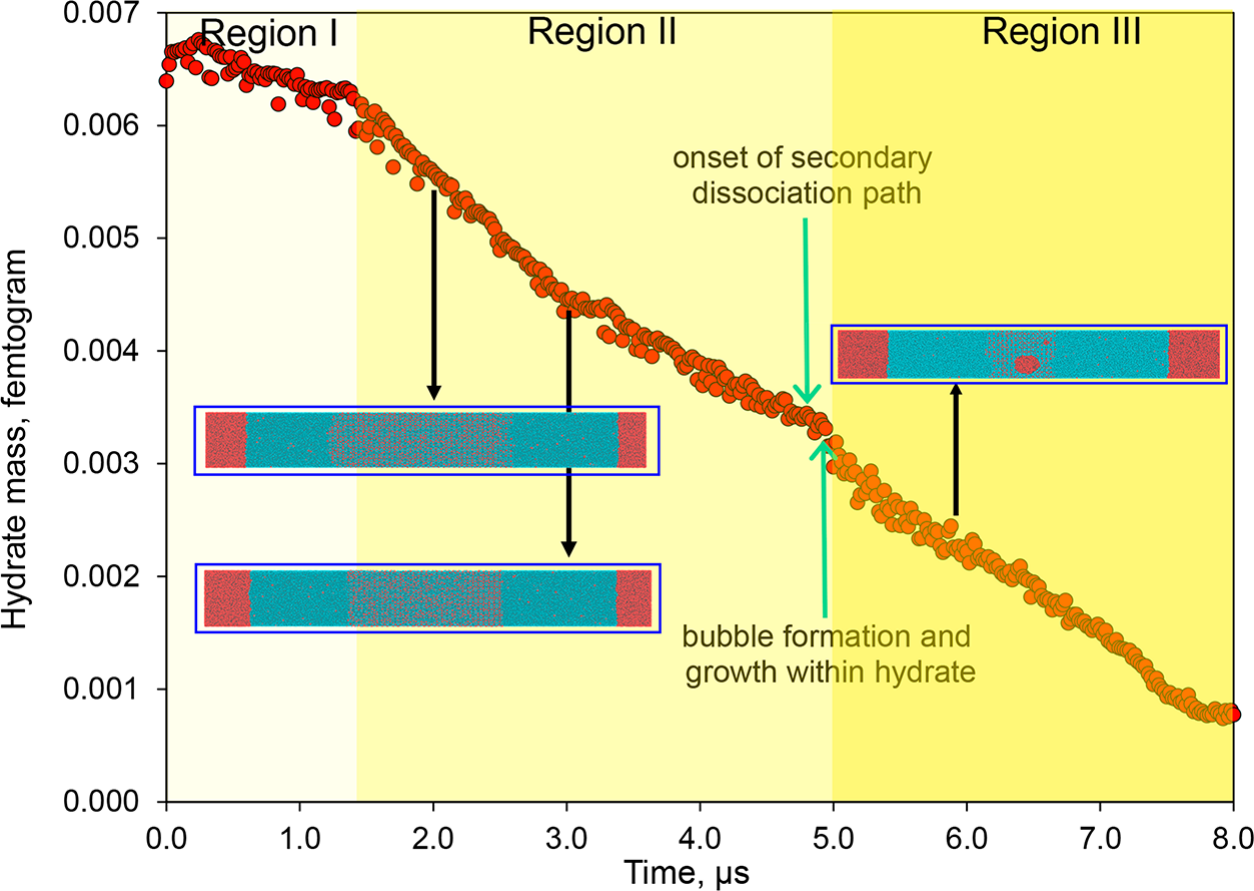
Mass of the remaining hydrate plotted against the simulation time for boundary temperatures of 288 K. The molecular images of the simulation box are interspersed along the plot, visually connecting them to their corresponding simulation time.

Figure 6 presents the plot of the resulting mass versus the simulation time. By inspecting the slopes of the mass–time plots in Figure 6, we identified three distinct dissociation regions: Region I is where dissociation is initiated, Region II is where steady dissociation occurs, and Region III is where the final dissociation period happens. Region II is the longest of the three regions (∼3.5 μs) and is the primary focus because of the fairly constant slope, which indicates a steady dissociation. Region I has the lowest slope, or dissociation rate, of the three regions. This could be attributed to the need to overcome the melting activation energy and break the hydrogen bonds at the crystal/liquid interface at the onset of dissociation. Movies S1–S4 also show that the bubbles formed at the crystal/liquid interface serve as a source of methane, continuously feeding the hydrate cages with free methane molecules and curtailing dissociation. In Region II, these methane bubbles disperse into liquid water. The onset of the secondary dissociation path (discussed in Nonplanarity of the Solid/Liquid Interface and Formation of a Secondary Dissociation Path) marks the end of Region II.

The sharp increase in the dissociation rate observed at the beginning of Region III (at ∼5 μs) can be attributed to the formation of methane bubbles within the solid hydrate. However, this increased dissociation rate is unstable and eventually decreases over time. The subsequent decrease in the dissociation rate can be explained by the sudden decrease in the average temperature of the simulation box by ∼2 K at the corresponding time, as seen in Figure 3E. As a result, although the secondary dissociation path increases the rate of dissociation, this accelerated dissociation is counteracted by the temperature drop, which is caused by the liberation of large volumes of methane. The hydrate dissociation stops at the end of Region III because the average temperature drops below the equilibrium temperature, as discussed in Figure 3E. Figure S4 provides a similar result for the *T*_b_ = 293 K case.

## Conclusions

The simulation of transient thermal dissociation using the coarse-grained mW model for water enables the study of systems much larger than those of previous molecular dynamics (MD) studies. Using these large-scale MD simulations, we report a novel observation of the formation of a secondary dissociation path that leads to the formation of gas bubbles within a solid hydrate. To the best of our knowledge, the observation of a secondary dissociation path and of gas bubbles within a solid hydrate has never been reported during hydrate dissociation. This could be attributed to previous MD studies using much smaller simulation boxes, which may be limited by size effects. In addition to these qualitative observations, we studied the kinetics of transient thermal dissociation by estimating the rate of mass dissociation from nonequilibrium simulations of 96 × 12 × 12 nm simulation boxes. Our observations indicate that the kinetics of dissociation is controlled not only by the average temperature but also by the formation of methane bubbles near the interface, the nonplanarity of the crystal/liquid interface, and the generation of a secondary dissociation path.

## Data Availability

Many of the tools developed as part of this study are openly available in the “Thermal Dissociation” GitHub repository at https://github.com/UnconvRS/ThermalDissociation.

## Supporting Information Available

Analogous results for the *T*_b_ = 293 K dissociation simulations and links for the complete set of movies (Movies S1–S4) showcasing the time evolution of our molecular dynamics simulations (for best movie quality, see the MP4s below) (PDF)
Protein data bank (pdb) structure for the s_I_ unit cell (PDB)
Stillinger–Weber potential parameters used in LAMMPS simulations for s_I_ methane hydrate (TXT)
Movie S1: molecular interaction of methane bubbles with water and the crystalline hydrate at the crystal/liquid interface at the beginning of the *T*_b_ = 288 K dissociation (MP4)
Movie S2: *T*_b_ = 288 K dissociation of the methane hydrate motifs at the crystal/liquid interface for the time interval 3.7–4.0 μs (MP4)
Movie S3: formation and growth of methane bubbles within the solid hydrate initiated by the secondary dissociation path for five *xy*-slices at *T*_b_ = 288 K (MP4)
Movie S4: the molecular events that led to the formation of gas bubbles within the solid hydrate at the third *xy*-plane, *T*_b_ = 288 K (MP4)

## Supplementary Material

jp3c03391_si_001.pdf

jp3c03391_si_002.pdb

## Abbreviations

MHRs: methane hydrate reservoirs
CGMD: coarse-grained molecular dynamics
mW: monatomic water
UA: united atom
CCS: CO_2_ capture and storage
NGH: natural gas hydrate
LAMMPS: Large-scale Atomic/Molecular Massively Parallel Simulator
SW: Stillinger–Weber
MD: molecular dynamics
VMD: Visual Molecular Dynamics
