# Supplementary material for: Large-Scale Nonequilibrium Molecular Studies of Thermal Hydrate Dissociation
Source: J Phys Chem B. 2023 Jul 18;127(29):6543–50. doi: 10.1021/acs.jpcb.3c03391 (PMC11008782; doi:10.1021/acs.jpcb.3c03391)
Supplement: Supplementary file 1 — jp3c03391_si_001.pdf [file jp3c03391_si_001.pdf]

Supplementary Information for:

Large-Scale Nonequilibrium Molecular Studies of Thermal Hydrate Dissociation

Meisam Adibifard[1] and Olufemi Olorode[1*]

Department of Petroleum Engineering, Louisiana State University; Baton Rouge, LA, United States

*Olufemi Olorode

Email: folorode@lsu.edu

This PDF file includes:

Supplementary text

Figures S1 to S4

Movies S1 to S4

Computational Resources

Considering that the coarse-grained MD simulations performed in this work are extremely computationally expensive, we leveraged the GPU implementation of LAMMPS in all simulations performed. We used the GPU-enabled high-performance computers (HPCs) available at the Center for Computation and Technology (CCT) at Louisiana State University (LSU) and obtained a performance of ~150 ns/day. The specifications of these HPCs are summarized below:

1. **SuperMike III:** This is a 1,285 Tflops HPC with 183 CPU nodes (each node has 64 2.6 GHz cores) and 8 GPU nodes (each has 4 NVIDIA A100 GPUs). The standard nodes have 256 GB RAM, whereas the big memory nodes have 2 TB RAM.

2. **QB3:** This is an 857 Tflops HPC with 202 CPU nodes (each node has 48 2.4 GHz cores) and 8 GPU nodes (each has 2 NVIDIA Volta V100 GPUs). The standard nodes have 192 GB RAM, whereas the two big memory nodes have 1.5 TB RAM.

Supporting Information Text

Figure S1 presents the corresponding transient thermal dissociation results for the case where T_b = 293 K. Figure S1(A) shows that after 0.38 μs, some of the methane gas molecules released coalesce to form bubbles relatively close to the solid/liquid interface. The images in Figure S1(B) and (C) show that the gas bubbles grow in size and agglomerate as more gas is released. Compared to the 288 K case, the temperature in the hydrate region of the simulation box first rises above the equilibrium temperature (Figure S1(A)). However, it stabilizes at the equilibrium temperature of 283 K after ~0.75 μs (Figure S1(B)).

Figure S2 presents the observation of the secondary dissociation path followed by the formation and growth of the gas bubble in the solid hydrate at T_b = 293 K. Although the progressions of these physical phenomena are similar to those in the 288 K case, these processes were observed much earlier (between 0.57 and 0.8 μs).

To assess the accuracy of the template-matching algorithm, we visually counted the number of unit hydrate cells in all five slices of the simulation domain (as in Figure 4) at five time steps (corresponding to 0, 25, 50, 75, and 100% of the total simulation duration). We then calculated the relative error of the template-matching algorithm compared to the visually counted data. The results are summarized in Figure S3, which shows that the absolute error of the template matching algorithm is accurate within 5% for four of the five points and less than 9.51% in the time step with the most error.

Figure S4 presents the plot of the remaining hydrate mass against the simulation time for the case with T_b = 293 K. We identified three distinct dissociation regions. Dissociation is initiated in Region I, steady dissociation occurs in Region II, and Region III shows the final dissociation period. The annotations of this figure indicate the onset of the secondary dissociation path and the gas bubble formation within the solid hydrate.

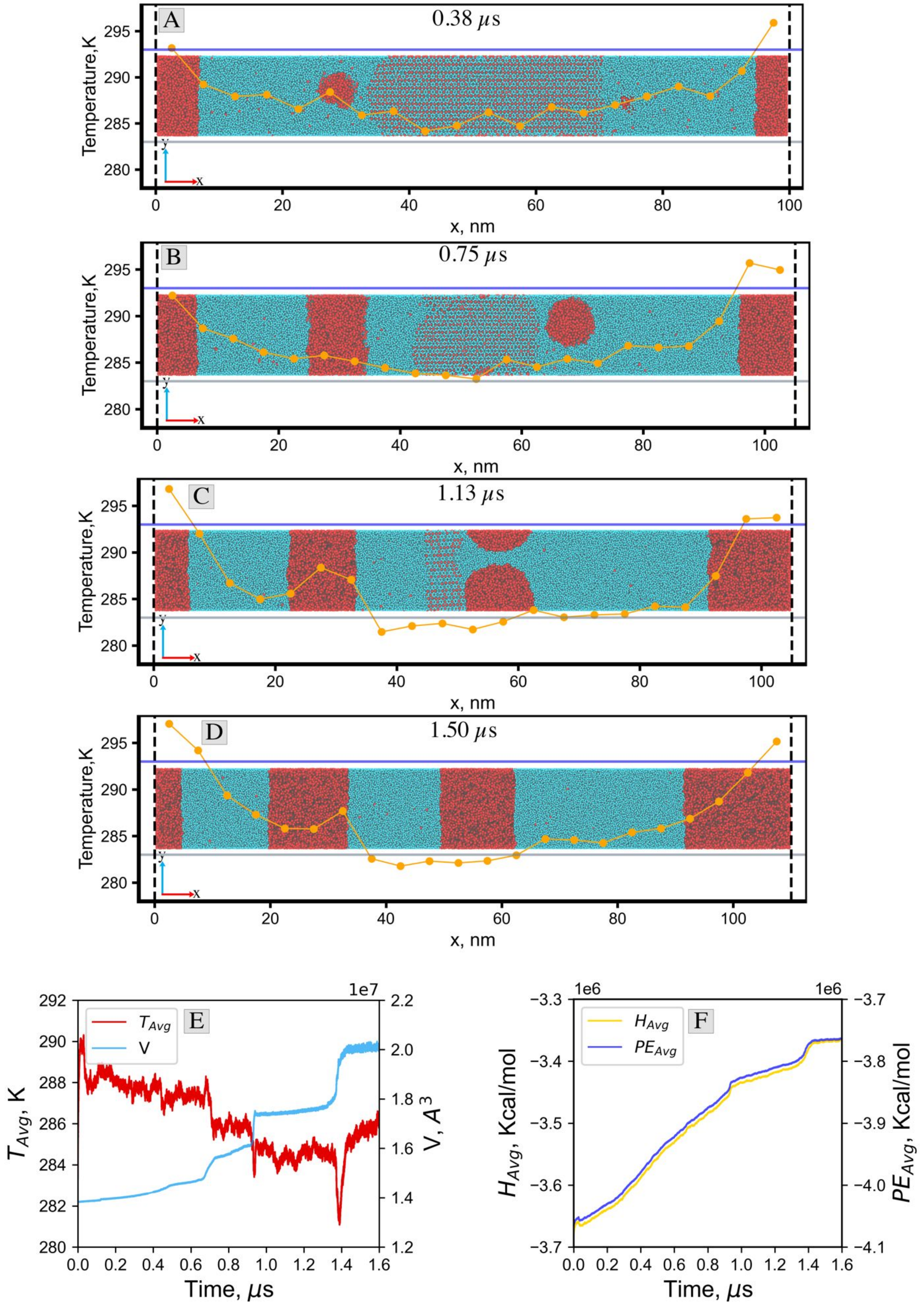

A
0.38 μs
B
0.75 μs
C
1.13 μs
D
1.50 μs
Temperature,K
x, nm
295
290
285
280
0
20
40
60
80
100
y
x
E
T_Avg
V
T_Avg, K
V, A^3
1e7
Time, μs
F
H_Avg
PE_Avg
H_Avg, Kcal/mol
PE_Avg, Kcal/mol
1e6
Time, μs

Figure S1. The snapshots of the molecular trajectories overlaid with the temperature profile and evolution of the average temperature, volume, enthalpy, and potential energy (PE) of the system during the nonequilibrium dissociation simulations at T_b=293 K. The snapshots represent the state of the system where (A) 25%, (B) 50%, (C) 75%, and (D) 100% of the simulation time. Water and methane are represented by red and cyan spheres, respectively. The orange line represents the temperature profile, and the blue and gray horizontal lines represent the boundary and equilibrium temperatures (T_b=293 K and T_{eq}=283 K). The dotted vertical lines indicate the boundaries of the box. Beyond the interface, the temperature rises gradually towards to boundary temperature of 293 K. The average temperature undergoes multiple sharp drops associated with abrupt increases in box volume towards the end of the simulation (E). (F) shows that the enthalpy and PE of the system increase steadily.

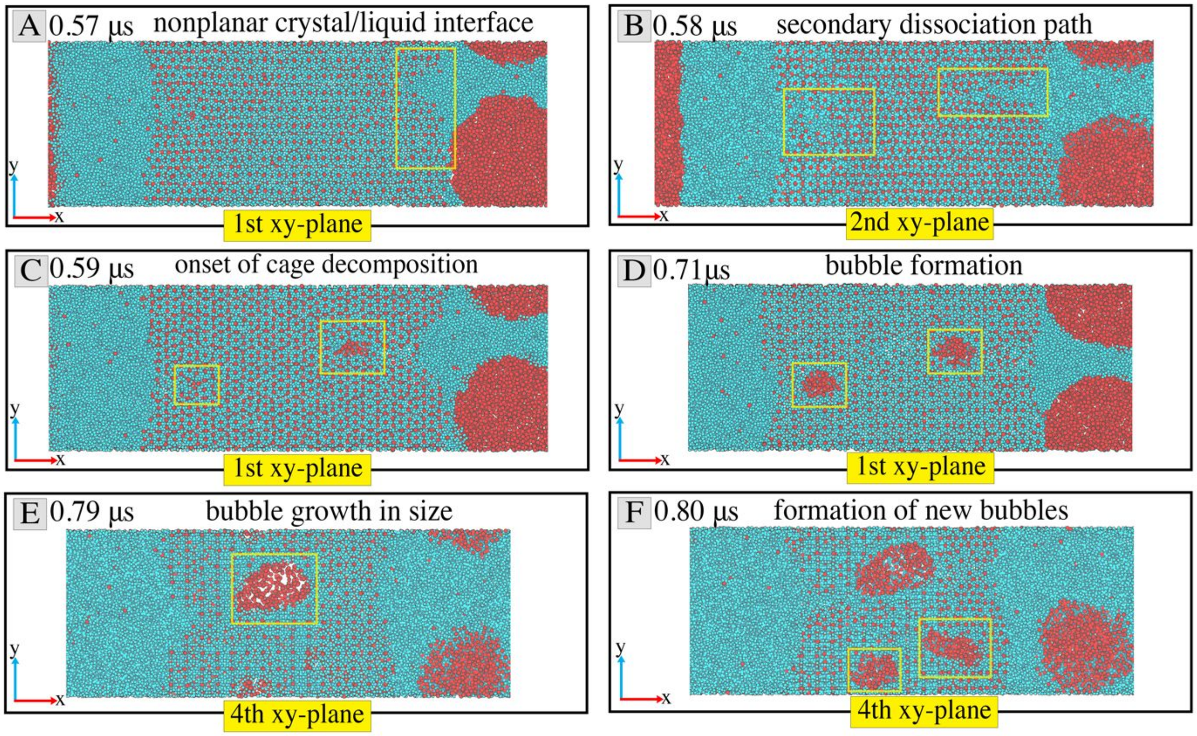

Figure S2. Trajectory snapshots illustrating the (A) nonplanarity of the dissociation front, (B) formation of the secondary dissociation path, (C) onset of cage decomposition within solid hydrate, (D) bubble formation in solid hydrate, (E) bubble growth in size, and (F) increases in the number of bubbles in hydrate, for T_b=293 K. The cyan and red spheres represent water and methane molecules, respectively.

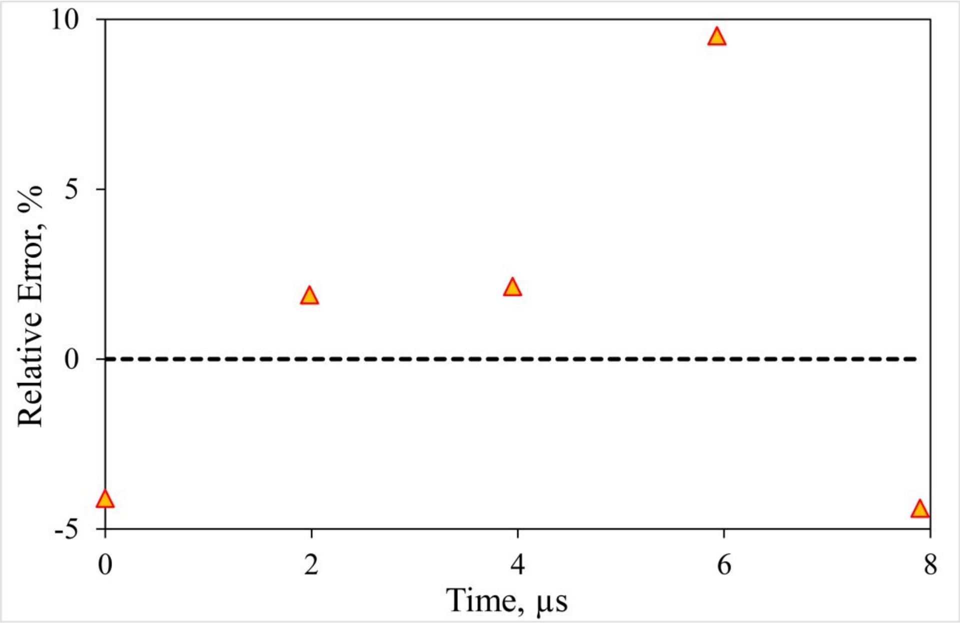

Figure S3. The relative error of the template-matching algorithm in counting the number of s_I unit-cells at five different simulation times where 0, 25, 50, 75, and 100% of the simulation is progressed. Errors are calculated compared to the visually counted number of unit cells.

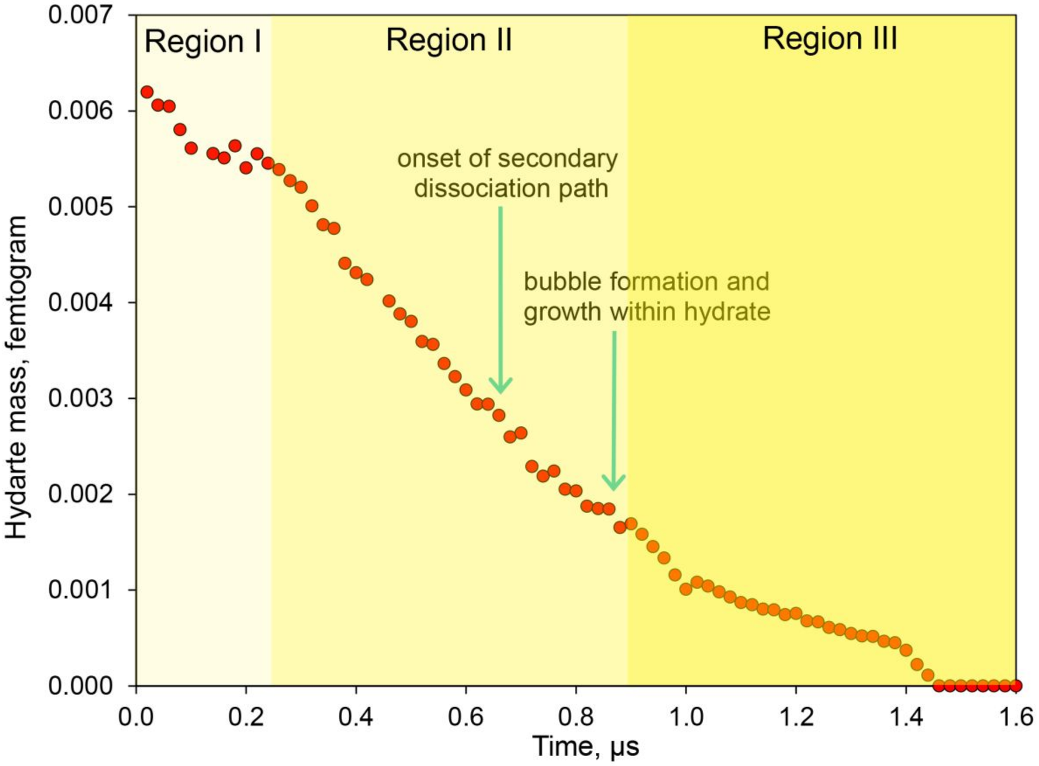

Figure S4. The remaining mass of hydrate against the simulation time for thermal dissociation simulations at T_b=293 K.

Movie S1. The linked movie shows the molecular interaction of the methane bubble with water and crystalline hydrate at the crystal/liquid interface at the beginning of the dissociation simulations at T_b=288 K. It provides a closer look into the crystal/liquid interface at the beginning of the dissociation (t ~ 0.3 ms). The water molecules (cyan spheres) that form the cages at the interface are continuously exchanged with the free water molecules from the liquid phase due to the breaking and reforming of the hydrogen bonds. Although this allows the trapped methane (red spheres) to escape, the cage keeps its structural integrity by instant enclathration of another free methane molecule.

Movie S2. The linked movie shows the dissociation of the methane hydrate motifs at the crystal/liquid interface for the time interval between 3.7 and 4.0 µs (the middle of Region 2 in Figure 6 of the manuscript) at T_b=288 K. The results show that the hydrate cages at the crystal/liquid interface undergo a continuous cycle of dissociation and reformation before they fully dissociate. Red and cyan spheres represent methane and water molecules, respectively.

Movie S3. The linked movie illustrates the formation and growth of the methane bubbles within the solid hydrate initiated by the ephemeral secondary dissociation path. Molecular trajectories are provided for five different xy-slices along the z-direction at a boundary temperature of 288 K. Methane and water are shown by red and cyan colors, respectively.

Movie S4. The linked movie provides a detailed look at the molecular events leading to the formation of gas bubbles within the solid hydrate at the third xy-plane. The hydrate's rigid movements cause the bubble to disappear and reappear intermittently. Methane and water are shown in red and cyan colors, respectively.
